# Single Versus Double Intramedullary Fixation of Paediatric Both Bone Forearm Fractures: Radiological Outcomes

**DOI:** 10.7759/cureus.2544

**Published:** 2018-04-28

**Authors:** Elizabeth A Crighton, James S Huntley

**Affiliations:** 1 Orthopaedics, Glasgow Royal Infirmary; 2 Department of Surgery, Sidra Medicine

**Keywords:** paediatric forearm fractures, elastic intramedullary nails

## Abstract

Introduction

Both bone diaphyseal forearm fractures are common in children. If the reduction is unstable, intramedullary fixation using elastic intramedullary nails (EIN) is an option. Intramedullary fixation may be either of single (S-EIN) or both (double) bones (D-EIN). Some reports have shown poorer outcomes with S-EIN. Our aim was to critically analyse the radiological features of EIN cases, comparing results for single and both bone fixation.

Method

Retrospective review (two years: November 2014–November 2016) of EIN forearm procedures. Radiological reduction of the radius/ ulna (AP/lateral) was measured on theatre fluoroscopy and six week radiographs. The results were categorised by angulation: (i) <10°, (ii) 10–20°, and (iii) >20°.

Results

Of 36 patients (19 boys, 17 girls), 13 had S-EIN (mean age 9.6 years, range 7–14) and 23 had D-EIN (mean age 10 years, range 7–14). In the S-EIN group, two and 11 had the ulna and radius fixed, respectively. Intraoperatively, of the 13 S-EIN patients, nine had <10° angulation of the radius or ulna, two had 10–20°and two had >20°. All 23 D-EIN patients had intraoperative radiology showing <10° angulation of both bones and maintenance of reduction of <10° angulation at six weeks post surgery. However, some S-EIN patients had increased deformation: at six weeks, four patients had 10–20° and three patients >20°. No patient in either group had revisional treatment. Time in cast postoperatively was similar in both groups: S-EIN, 6.15 weeks (4–12) and D-EIN, 5.5 weeks (3–8). Operative time was 64 mins (43–82) and 76 mins (45–86) in S-EIN and D-EIN groups, respectively. No other complications were recorded.

Conclusion

Though there may be particular reasons for selecting single bone fixation, this series shows a propensity to increased angulation of fractures fixed by S-EIN (7/13 in this group). We advise caution in the use of single bone fixation for both bone forearm fractures.

## Introduction

Forearm fractures are common in children, accounting for 40% of all fractures, with 5.4% of these being of both bone diaphyses [1], thus forming a substantial proportion of the orthopaedic workload. Management of this injury depends on various features including age of the child, angulation/translation of the fracture, and stability of the reduction. The majority of reduced forearm fractures can be treated with cast immobilisation because of the strong periosteum and remodelling potential [2-5]. Relative indications for surgery of diaphyseal fractures include open injury, 'floating elbows', instability after reduction, and irreducibility (by closed means) [4-7].

Intramedullary fixation of paediatric forearm fractures with elastic stable intramedullary nail (EIN) can produce excellent outcomes [8-11]. EIN provides relative stability in the femur and tibia by trifocal internal buttressing, the two nails being maximally separated at the fracture site [12]. However, the smaller diameter of the radius and ulna mean only one EIN can be passed, acting as an intramedullary splint rather than a trifocal buttress. Our study aims to compare the radiological outcomes of single versus double bone intramedullary fixation.

## Materials and methods

Study design

This was a single centre retrospective review of children (less than 16 years of age at the time of injury) undergoing EIN for displaced unstable diaphyseal forearm fractures, between November 2014 and November 2016. Only patients with unstable both bone complete diaphyseal fractures (including, but not confined to, refractures and open injuries) treated by EIN were included. Previously published criteria for acceptable reduction were used [3-5, 13]: an angulation of more than 10° of the shaft was defined as suboptimal (unacceptable). Translation of greater than half the diameter of the bone and any rotation were deemed unacceptable.

Procedure

A reduction was performed in theatre under general anaesthesia with intraoperative fluoroscopy. Once optimal reduction was achieved the fracture was assessed for stability. Decision-making and procedure were performed by a consultant paediatric orthopaedic surgeon. If the reduction was stable, a moulded cast was applied; conversely, if unstable and/or an acceptable reduction not maintainable in cast, then EIN (this series) or another mode of fixation (excluded from this series) was used. The decision to use single (S-EIN) or double EIN (D-EIN) was at the operating surgeon's discretion based on stability and adequacy of reduction after first bone fixation. Generally, the more substantially displaced bone was fixed first. Either EIN (2 mm/2.5 mm) or stainless steel K wire was used.

Follow-up

All patients underwent clinical and radiological review at two and six weeks after operation, with further follow-up until union and EIN removal.

Outcome

The primary outcome was diaphyseal angulation (radius and ulna) on the six week post operation radiograph. This was defined as the largest angulation on the anteroposterior and lateral views. Secondary data included requirement for open reduction, surgical time, time in cast, time until EIN removed, and complications.

Research approval

Approval for this study was sought and achieved from our institute research and development department and the  reference number is GN 18OR129.

Statistical analysis

Where appropriate, data are given as mean or median, with range.

## Results

Between November 2014 and November 2016, 36 children (19 boys, 17 girls; mean age 10 years) underwent EIN for both bone diaphyseal fracture. Patient characteristics and seminal features for the two groups are summarised in Table 1.

**Table 1:** Characteristics of patients undergoing EIN for both bone diaphyseal fractures EIN - elastic intramedullary nail

Characteristic	Single-EIN	Double-EIN
Number of patients	13	23
Age, years	9.6 (7-14)	10 (7-14)
Sex, Female:male	6F:7M	11F:12M
Open fracture	2	3
Refracture	3	1
Open reduction	1	6
Operative time (minutes)	64 (43-82)	76 (45-86)
Time in cast (days)	43 (28-84)	38 (21-56)
Time until EIN removed (weeks)	21.5 (5-48)	23.1 (9-52)
Complication (other than angulation)	0	0

All sustained the injury from a fall onto an outstretched hand, most commonly involving a trampoline. Five were grade 1 Gustilo-Anderson open fractures. Four were re-fractures of a previously conservatively managed fracture. Thirteen patients had S-EIN (mean age 9.6 years, range 7–14) and 23 patients had D-EIN (mean age 10 years, range 7–14). Of the S-EIN patients, two were of the ulna and 11 of the radius. One patient in both S-EIN and D-EIN groups had the ulna fixed with an intramedullary K-wire rather than an EIN (i.e, only two patients had a K-wire used).

Operative time was 64 mins (43–82) and 76 mins (45–86) in S-EIN and D-EIN groups, respectively; one patient required open reduction for S-EIN and six patients for D-EIN. The average time in cast was 43 days (28–84) and 38 days (21–56) in S-EIN and D-EIN groups, respectively. All metalwork was buried, apart from in one patient, where it was left percutaneous and removed at five weeks in clinic. All other EIN were removed in theatre after union, at 21.5 weeks (5–48) for S-EIN and 23.1 weeks (9–52) for D-EIN.

No patient was lost to follow-up. There was no complication or reoperation performed in either group.

Radiographs were taken preoperatively, intraoperatively, and at six weeks postoperatively. The greatest angulation on the anteroposterior or lateral radiograph was measured. The angulations were grouped into (i) less 10° (adequate), (ii) 10–20° (borderline), and (iii) greater than 20° (inadequate). The angulation of the radius and ulna on preoperative radiographs is presented in Table 2.

**Table 2:** Preoperative radiograph angulation of radius and ulna EIN - elastic intramedullary nail

Variable	S-EIN Pre- op (degree)	D-EIN Pre- op (degree)
Ulna, AP Median (range)	18 (0-35)	19 (0-48)
Ulna, Lateral Median (range)	9 (0-20)	12 (0-40)
Radius, AP Median (range)	11 (0-27)	12 (0-26)
Radius, Lateral Median (range)	18 (0-32)	21 (8-41)

Both groups had similar preoperative angulation. Intraoperative and postoperative angulations differed between the groups and are displayed in Tables 3-4.

**Table 3:** Angulation of radius and ulna on double EIN intraoperative and postoperative radiographs EIN - elastic intramedullary nail

Intraoperative Fluoroscopy			6 week radiograph		
Angulation (degree)	Radius (no.)	Ulna (no.)	Angulation (degree)	Radius (no.)	Ulna (no.)
<10°	23	23	<10°	23	23
10-20°	0	0	10-20°	0	0
>20°	0	0	>20°	0	0

**Table 4:** Angulation of radius and ulna on single EIN intraoperative and postoperative radiographs EIN - elastic intramedullary nail

Intraoperative Fluoroscopy			6 week radiograph		
Angulation (degree)	Fixed bone	Non Fixed bone	Angulation (degree)	Fixed bone	Non Fixed bone
<10°	9	13	<10°	10	9
10-20°	2	0	10-20°	1	3
>20°	2	0	>20°	2	1

For D-EIN intraoperative radiography, both the bones were adequately reduced, i.e., all 23 patients had <10° angulation of both the radius and ulna. For S-EIN (n = 13) intraoperative radiography, the non-fixed bone (two radius and 11 ulna) was acceptably reduced with <10° angulation in all; however, the fixed bone (11 radius and two ulna) had >10° angulation in four patients.

At the six week postoperative radiography, the D-EIN group angulation remained the same, with all 23 patients maintaining <10° angulation of both the radius and ulna. However, patients in the S-EIN group had heterogeneous results: four of the non-fixed bones progressed from <10° intraoperative angulation to >10° angulation at six weeks.

There was no correlation between age and progressive postoperative displacement of the unfixed bone in the S-EIN, with an average age 9.3 years (7–12) compared to 9.8 years (5–14) in non-progressive fractures. All non-fixed bones that progressed were ulnas. Although the numbers are small, it was noted that preoperative displacement correlated to an increased chance of postoperative displacement if unfixed: preoperative displacement of the ulna in the patients that progressed was 24° (5–35°) in contrast to 14° (0–20°) in the static group.

The shape of the EIN may also have influenced progression of deformity; we noted that all four of the S-EIN patients who showed progressive deformity of the non-fixed ulna had a C-shaped EIN rather than S-shaped (see figures below).

## Discussion

Intramedullary fixation with EIN or (K-wires) is a popular and accepted method of treating unstable diaphyseal forearm fractures [6,9]. They provide more security against loss of position of an unstable fracture than a cast and offer smaller scars, shorter procedures, and equivalent functional results to rigid internal fixation [6]. Flynn and Waters were the first to describe the use of single bone intramedullary fixation of both bone forearm fractures [14]. They reported excellent functional results of nine patients who had single intramedullary nail placed within the ulna and one patient within the radius; eight patients regained full range of movement and two lost 5° of pronation. Several small studies have reported similar results [15,16]. The largest study included 49 patients and compared S-EIN to D-EIN, finding no difference in functional results or complications between the two groups [2].

Some literature, however, has reported concerns about S-EIN, particularly in older children and when the ulna is fixed alone. A multicentre randomised control trial comparing S-EIN to D-EIN in 24 patients reported that S-EIN led to increased redisplacement and reduced clinical results [13]. Dietz et al. compared D-EIN with S-EIN fixation of the ulna [8], finding it to be 'safe and efficacious' in younger children, but to be used with caution in older children or open fractures, due to increased redisplacement. Due to the dubiety in the literature, our study aims to further analyse S-EIN versus D-EIN in paediatric both bone diaphyseal forearm fractures.

Despite previous studies showing reduction in operative time with S-EIN, this was not substantially different from D-EIN (S-EIN 64 mins; D-EIN 76 mins). The time in cast postoperatively was marginally longer in the S-EIN group (S-EIN 43 days; D-EIN 38 days), but noticeably, one patient was in cast for 12 weeks due to concerns over progressive displacement of the fracture. All the fractures united and no complications were reported. No patient required revision surgery at two years after EIN.

**Figure 1:**
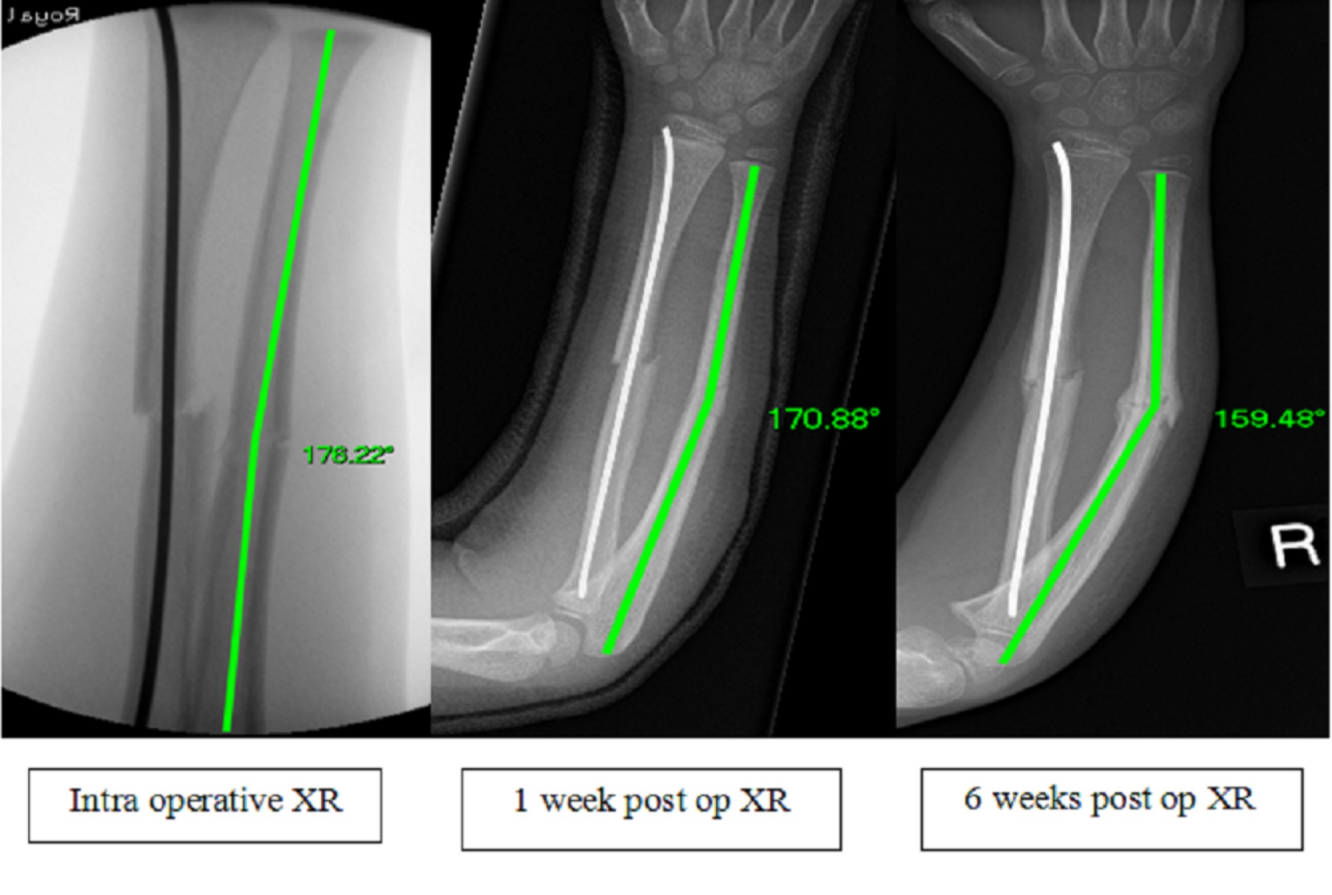
Intraoperative, 1 week and 6 week postoperative radiographs of S-EIN to radius with progressive deformity of ulna over time. Shown with on-screen angle measurement. S-EIN - Single elastic intramedullary nail

The 23 patients undergoing D-EIN all had <10° angulation at both intraoperative and six week postoperative radiographs. There was no progression in deformity and no complications were recorded. We report substantially poorer radiological outcomes in our S-EIN group; seven out of 13 patients had >10° angulation of either the fixed, unfixed or both bones on the six week postoperative radiograph. Despite the small numbers in our study, there appeared to be two distinct groups within the S-EIN cohort: (i) the fixed bone was never satisfactorily reduced and despite the non-fixed bone being adequately reduced in theatre, it showed progressive deformity over time (four out of 13 patients), (ii) adequate reductions of both bones in theatre but the non-fixed ulna had progressive subsequent deformity (Figure 1).

In certain situations, S-EIN may be an acceptable technique; however, we contend that generally D-EIN is safer as regards minimising the chance of progressive deformity. S-EIN should be avoided when the bones cannot be anatomically reduced.

When trying to ascertain factors to identify this group preoperatively, there appeared no correlation between age, open fracture or open reduction with progressive postoperative deformity. The factor suggestive of subsequent drift for S-EIN was substantial preoperative angulation of the ulna; in those that progressed, this was mean (range), 24° (5–35°), in comparison to 14° (0–20°) for non-progression of non-fixed ulna. In addition, none of the patients that had S-EIN for a refracture of a previously conservatively managed fracture had subsequent progression of the non-fixed bone.

**Figure 2:**
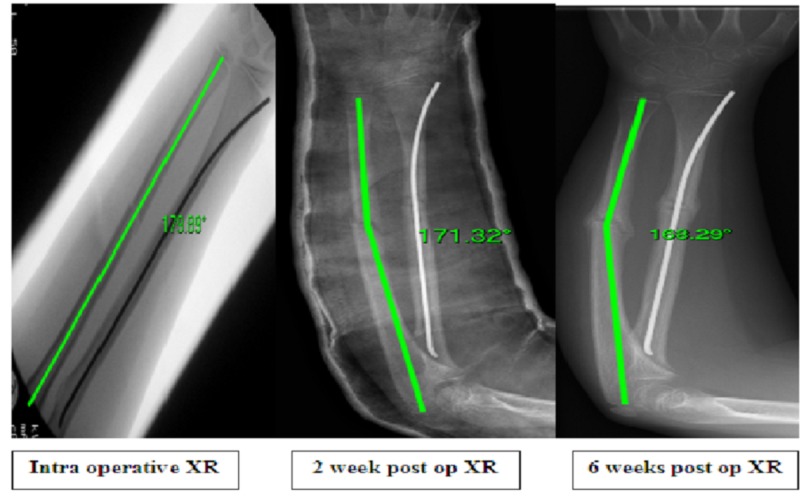
Intraoperative, 2 week and 6 week radiographs of patient with S-EIN of radius and postoperative deformity of the ulna. Shown with on-screen angle measurement. S-EIN - Single elastic intramedullary nail

**Figure 3:**
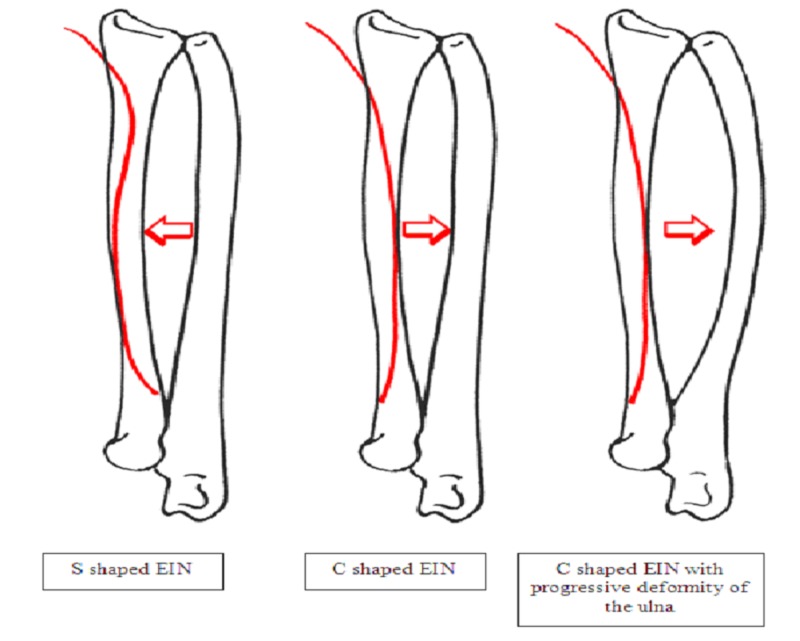
Representation of S-shaped EIN restoring radial bow and C-shaped causing deformity of the ulna. EIN - elastic intramedullary nail

Schemitsch and Richards speculated that restoration of the radial bow was the key to good reduction and securing the function of forearm rotation [17]. Although we only measured angulation in our study, the general consensus is that angulation of >10° causes rotational loss [18]. Due to EIN being inserted just proximal to the radial styloid it has the propensity to act against the radial bow unless prebent in an 'S' shape rather than a 'C' [19] (Figures 2-3). Prevention of the EIN displacing the radial bow may prevent progressive deformity in the non-fixed ulna.

## Conclusions

In conclusion, our study details results from single and double bone fixation with elastic stable intramedullary nails for both bone diaphyseal forearm fractures in children. We advise caution in the use of single bone fixation of both bone fracture due to the propensity to increased angulation and progressive deformity. In certain patients, S-EIN might be an acceptable treatment option but it is vital that the fixed bone is anatomically reduced, with particular attention paid to the radial bow. If fixation is required, double bone fixation is generally a construct associated with better outcomes.
